# Nutrients and Dementia: Prospective Study

**DOI:** 10.3390/nu15040842

**Published:** 2023-02-07

**Authors:** Hikaru Takeuchi, Ryuta Kawashima

**Affiliations:** 1 Division of Developmental Cognitive Neuroscience, Institute of Development, Aging and Cancer, Tohoku University, Sendai 980-8575, Japan; 2 Smart Aging Research Center, Tohoku University, Sendai 980-8575, Japan; 3 Department of Advanced Brain Science, Institute of Development, Aging and Cancer, Tohoku University, Sendai 980-8575, Japan

**Keywords:** nutrients, dementia, magnesium, protein, sugar

## Abstract

The association of diet and nutrients with dementia risk is an interesting research topic. Middle-aged and older Europeans not diagnosed with dementia within two years of baseline were followed up and their data were analysed until 2021. The association between the nutrient quintiles measured by the web-based 24 h dietary and the risk of developing dementia was examined using a Cox proportional hazard model after adjusting for potential confounding factors. Approximately 160,000 subjects and 1200 cases were included in the analysis of each nutrient. A greater risk of dementia was associated with (a) no alcohol intake (compared with moderate to higher intake), (b) higher intake of total sugars and carbohydrates (compared with lower intake), (c) highest or lowest fat intake (compared with moderate intake), (d) quintiles of highest or lowest magnesium intake (compared with the quintile of the second highest intake), and (e) highest protein intake (compared with moderate intake). Overall, the present results are congruent with the importance of a moderate intake of certain nutrients.

## 1. Introduction

The increasing incidence of dementia is an important social issue in this current ageing society. No effective treatment has been developed for dementia; thus, the preventive effects of various lifestyle factors, including nutrition and diet, are currently being investigated as they hold great importance. Numerous cohort studies have been conducted on the association between the intake of different nutrients and the risk of dementia. In addition, meta-analyses revealed that a higher risk of dementia is associated with a lower intake of unsaturated fatty acids [1], folate [2], vitamin D [1], vitamin E [3], and minimal or no alcohol intake [4]. However, some reports are inconsistent (e.g., findings on vitamin E) [5]. In addition, the association between the lower risk of dementia and the intake of magnesium, proteins, other nutrients, and some forms of sugars was observed only in individual observational studies using a relatively smaller sample size than those using UK Biobank data [6,7,8,9].

These observational studies have various problems. First, they were generally small in size (compared with those of UK Biobank studies). Although meta-analyses can compensate for this limitation, they are not free from publication bias. Second, the abundant potential confounding factors have a potential impact. For instance, our previous study showed that body mass index (BMI) and the risk of dementia are significantly affected by adjusting for educational history [10]. Although previous meta-analyses linked obesity in middle age to a higher risk of dementia [11], recent large studies found the opposite effect [12,13]. The underlying reason is uncertain and may be partly due to the increase in effective coping strategies for stroke. Thus, investigating relevant associations using modern data is important.

To address these issues, we used data from the UK Biobank to reveal the association between dietary nutrients and the risk of dementia in a large cohort after adjusting for a wide range of confounding factors. Our hypothesis was that a lower risk of dementia is associated with a higher intake of polyunsaturated fatty acids, protein, vitamins B, D, and E, and magnesium; a moderate alcohol intake; and a low sugar intake. We also conducted an exploratory investigation of nutrients and dementia risk. The increasing incidence of dementia is an important issue for the modern ageing society, and the identification of dietary habits associated with its prevention and risk is an important scientific topic. Further, the strengths of this study are summarized as follows: First, the large sample and careful adjustment for confounding factors attempt to provide robust answers to an important research topic (nutrition and dementia risk) on which previous findings have been mixed. Second, this study aims to confirm the findings in a modern sample. Finally, we are investigating nutrients not well investigated previously.

## 2. Methods

### 2.1. Participants

The UK Biobank provided a dataset obtained from a prospective cohort study of a middle-aged population in the United Kingdom [http://www.ukBiobank.ac.uk/wp-content/uploads/2011/11/UK-Biobank-Protocol.pdf, (accessed on 5 July 2021)]. The North–West Multi-Centre Research Ethics Committee approved these experiments, and each participant provided written informed consent.

The online dietary survey was administered five times between 2009 and 2012. The data of subjects who participated in the survey at least once were used in the analysis. For subjects who participated in the survey more than once, the average of each data type was used. A total of 211,013 subjects participated in the online dietary survey at least once.

In addition, each participant attended to one of the 22 assessment sites in the United Kingdom for data collection; baseline data were received from 502,505 participants in this cohort. Our analysis also included data from the first assessment visit (2006–2010) for the covariates of the analysis. We conducted each analysis using data from all participants for whom valid data for all independent and dependent variables were available.

The descriptions in this subsection are largely reproduced from our previous study using data from the UK Biobank [14].

### 2.2. Assessment of Nutrients

Nutrient item data were derived from the web-based 24 h dietary assessment “Oxford WebQ” [15]. The questionnaire was administered from 2009 to 2012. Oxford WebQ contains questions on the consumption of 206 foods and 32 beverages over the past 24 h. Subjects enlisted in the last year of the UK Biobank’s subject recruitment list were not enrolled through the web but rather through their email information when they were invited to participate in the study and took the survey 1–4 times between 2009 and 2012. The nutrients were calculated from the intake frequency, standard portion size, and nutrient composition of each food and beverage type. For subjects who took the survey more than once, the average value was used.

### 2.3. Sociodemographic and Lifestyle Measurements as Covariates

Self-reported gender data were used. From the UK Biobank database, the neighbourhood-level socioeconomic status at recruitment (cov1), education level at recruitment (cov2), household income at baseline (cov3), employment status at baseline (cov4), metabolic equivalent of task hours (MET) (cov5), number of people in the household (cov6), height (cov7), BMI category (cov8), self-reported health status (cov9), category of duration of sleep (cov10), category of diastolic blood pressure (cov11), current tobacco smoking level (cov12), ethnicity (cov13), diagnosis of diabetes, heart attack, angina, stroke, cancer, and other serious medical conditions (cov14–cov19), visuospatial memory task performance (number of errors: performance worse than 2SD were excluded) (cov20), depression score (cov21), antihypertensive medication (cov22), and statin use (cov23) were extracted or calculated and included as covariates together with age and sex. Additional information can be found in the Supplemental Methods.

The reason for including covariates for disease and health status in the analysis is to prevent, as far as possible, health status from being a confounding factor in the association between dementia and nutrients. That is, poor health leads to certain eating habits, such as eating small meals, and poor health is a risk for dementia, and the association between nutritional intake and dementia as a result of these two factors is prevented as much as possible by this model. Height was also included as a covariate to avoid the possibility of height being a confounding factor in the association between dementia and nutrients, as height is related to nutrient intake but also to dementia risk [16].

When all explanatory variables were treated as continuous variables and the correlation coefficients between explanatory variables calculated, the single correlation coefficient r was >|0.5| for the association between sex and standing height, and the association between age and current employment status and these associations did not include nutritional variables. Thus, multicollinearity did not appear to affect the association between nutritional variables and dementia. To confirm this, we excluded standing height and current employment status from the covariates and found that the adjusted hazard ratios of each group of nutrient intake level were barely affected and the significance (FDR-corrected) of the overall group differences was not affected.

### 2.4. Statistical Data Analysis

Predictive Analysis Software version 22.0.0 (SPSS Inc., Chicago, IL, USA; 2010) was used for statistical analyses. Cox proportional hazard models were used to investigate the relationship between diet type and the risk of all-cause dementia over time [17]. All-cause dementia was determined using hospital inpatient records and connections to death registry data. Additional information can be found in the Supplemental Methods. This method of determining dementia was adopted from a representative study that assessed lifestyle and risk of incident dementia over time using UK Biobank data [17] and from our previous work [14]. The descriptions in this subsection are largely reproduced from our previous study using the same methods [14].

Exclusion criteria were as follows: (a) self-reported dementia, Alzheimer’s disease, or cognitive impairment without a diagnosis of all-cause dementia in either hospital inpatient records or death register data; (b) a diagnosis of dementia at baseline or within two years after providing the answer to the first diet type question; (c) death within two years after baseline; and (d) visuospatial memory performance <2SD. The observation period started when each participant had first completed the diet type questionnaire and continued until death, dementia diagnosis, or until 30 September 2021. For each analysis, sex, age at completion of the first diet type questionnaire, and cov1–cov24 values were all used as covariates. People who developed dementia within two years were excluded from the analysis to eliminate the possibility that certain behaviour patterns are already being observed as a result of dementia. This approach has been previously applied in other studies on dementia [18].

Results with a *p* < 0.05 corrected for false-discovery rates using the two-stage sharpened method [19] in the analyses of group differences in each nutrition type were considered statistically significant. This correction was applied to the *p* values of the 23 main analyses of group differences for each type of nutrition.

In this study, we included subjects with complete data of covariates and nutritional data among those not excluded according to the four exclusion criteria of dementia. The UK Biobank, like any cohort study, involves participants with specific characteristics; therefore, it is likely that the subjects who took part in the online dietary survey also have specific characteristics. Moreover, participants in the online dietary survey, for whom complete covariates’ data are available, may also have specific characteristics. However, this study is an analysis within those specific cohorts, and all samples have the same conditions; in that respect, the removal of participants with a lack of data is unlikely to be a confounding factor (however, the generalizability of the results or their sensitivity may be altered). Among the participants in the UK Biobank not excluded according to the four exclusion criteria of dementia that completed the online survey and had nutritional data, the subjects who lacked ≥1 covariate (those excluded in the analysis) tended to be systematically different from those with complete covariate data. The former is particularly characterized (effect size: Cohen’s d > 0.3 or odds ratio >1.3 or <0.7) by low education level, low household income, non-current employment, female sex, non-white ethnicity, doctor diagnosis of diabetes, and doctor diagnosis of angina. These data are provided in Supplemental Table S1.

## 3. Results

### 3.1. Basic Baseline Data

Table 1 shows the baseline psychological variables of the participants. A total of 161,376 subjects participated in the online dietary survey, provided all data used in the analysis, and failed to meet the exclusion criteria. Among them, 160,170 (mean age of 58.5 [SD: 8.0] years) did not develop dementia, and 1206 (mean age of 66.3 [SD: 5.2] years) developed dementia.

### 3.2. Prospective Dementia Analysis

A total of 211,013 subjects participated at least once in the online diet survey. Among them, 28 who had a record of only self-reported dementia or cognitive impairment, 53 who had dementia diagnosis before baseline, and 69 who were diagnosed with dementia within two years after their last participation in the online dietary survey were excluded. A total of 1431 participants who died without a dementia diagnosis within two years after baseline were also excluded. Analyses were conducted using only the data of subjects who had all covariates, including those with visuospatial memory performance >2SD.

A Cox proportional hazard model split the subjects into five categories according to the nutrient intake level (mostly quintiles) to correct for a wide range of potential confounding factors. A correction for multiple comparisons was conducted as well. One analysis per nutrient was performed for a total of 23. The results showed significant group differences for alcohol, fat, carbohydrate, protein, total sugars, and magnesium. However, no group differences existed for calcium, carotene, energy, energy dietary fibre, folate, food weight, potassium, polyunsaturated fat, iron retinol, starch, saturated fat, and vitamins B6, B12 C, D, and E. Statistical values and adjusted rates, as well as the number of cases and samples in each group in each analysis, are provided in Table 2.

**Figure 1:**
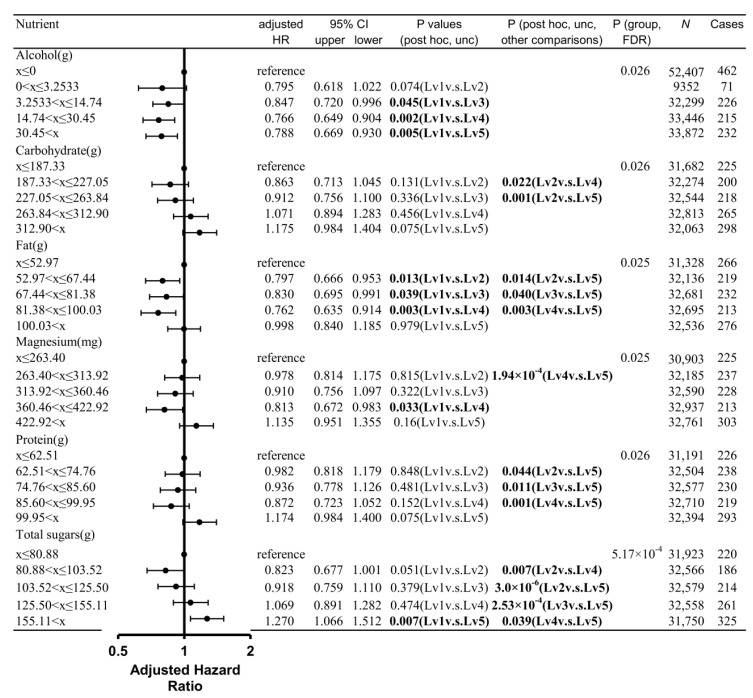
Standardized risks of incident dementia over time according to the intake level of each nutrient (alcohol, carbohydrate, fat, magnesium, protein, and total sugars). Cox proportional hazards models were adjusted for potential confounding variables. The adjusted hazard ratios of each intake level group, compared with the group with the lowest intake level and their 95% confidence intervals, are provided. The *p* values of overall group difference (p (group)) and post hoc comparisons between each group as well as the size of the entire group and the dementia cases included in each group are shown. Bold = *p* < 0.05.

For alcohol, post hoc analyses revealed that groups with intake levels 3–5 (highest intake levels) showed a significantly lower risk of dementia than that with intake level 1 (lowest intake level and no alcohol intake; Figure 1).

For carbohydrates, post hoc analyses revealed that groups with intake levels 4 and 5 (highest intake levels) showed a significantly higher risk of dementia than that with intake level 2 (second lowest intake level; Figure 1).

For fat, post hoc analyses revealed that groups with intake levels 2–4 (intermediate intake levels) showed a significantly lower risk of dementia than those with intake levels 1 (lowest intake level) and 5 (highest intake level) (Figure 1).

For magnesium, post hoc analyses revealed that the groups with intake levels 1 (lowest intake level) and 5 (highest intake level) showed a significantly higher risk of dementia than that with intake level 4 (second highest intake level; Figure 1).

For protein, post hoc analyses revealed that the groups with intake levels 2–4 (intermediate intake levels) showed a significantly lower risk of dementia than the group with intake level 5 (highest intake level) (Figure 1).

For total sugars, post hoc analyses revealed that the groups with intake levels 1–4 showed a significantly lower risk of dementia compared with that with intake level 5 (highest intake level; Figure 1). In addition, the group with intake level 4 (second highest intake level) showed a significantly higher risk of dementia than that with intake level 2 (second lowest intake level).

### 3.3. Sensitivity Analyses Controlling for Energy Intake Quintiles

In addition to all other covariates in the main analyses, the quintile of total energy intake was incorporated as a variable in the sensitivity analysis of each nutrient.

The results of the alcohol analysis showed a similar adjusted hazard ratio for each group and a significant *p*-value for the presence of overall group differences after corrections for multiple comparisons. The results for carbohydrates and total sugars show a larger adjusted hazard ratio for the higher intake groups, with strong significant differences.

The analysis for protein shows a slightly smaller adjusted hazard ratio for the highest intake group (1.17 ≥ 1.15) and a slightly larger adjusted hazard ratio for the second (0.87 ≤ 0.89), resulting in a small difference between the two. However, this change does not alter our discussion, as the difference between the two groups was still significant in the post hoc analysis (*p* = 0.008).

Similarly, the results for magnesium show a slightly smaller adjusted hazard ratio for the highest intake group, whereas that for the second highest intake group remains almost the same, resulting in a smaller difference between the groups. However, this change does not change our discussion, as the difference between the two groups is still significant in the post hoc analysis (*p* = 0.003).

Finally, the analysis for fat shows that the adjusted hazard ratio for the highest intake group substantially decreased (1.00 ≥ 0.81); the result of the energy intake-adjusted analysis shows almost an L-shaped relationship, with the lowest intake only indicating higher risk. Similarly, the adjusted hazard risk for the highest (0.89 ≥ 0.75) and second highest intake groups (0.74 ≥ 0.067) in the polyunsaturated fatty acid results decreased; the *p*-value for the presence of overall group differences was significant after multiple comparison correction. All statistical results are provided in Table 3.

## 4. Discussion

We used a large dataset of middle-aged and older people in the UK to examine the relationship between the intake level of various nutrients and the risk of developing dementia >2 years after baseline while adjusting for a wide range of confounding factors. The present results were partly consistent with our hypothesis; that is, a moderately high intake of basic nutrients, such as protein, and fat, is associated with a lower risk of incident dementia, and no alcohol intake is associated with a higher risk of dementia over time. In addition, we confirmed our hypothesis that a higher magnesium intake is associated with a lower risk of dementia. We generally confirmed the association between a higher intake of total sugars or carbohydrates and a greater risk of incident dementia. Meanwhile, polyunsaturated fat, folate, and vitamins D and E were not significantly associated with the risk of dementia over time, although some results showed a statistical tendency. Many studies have shown that people taking a little to a moderate level of alcohol have a lower risk of dementia than those taking no alcohol at all, though the dose–response relationship was L-shaped in some studies [20] and U-shaped in others [4]; this study could well replicate this finding. The replication of these findings seems to indicate the robustness of these and other findings (as discussed below).

The link between a higher magnesium intake and a lower risk of dementia in this study was partly consistent with previous research and may be due to the intrinsic properties of this nutrient. The second-highest magnesium intake group in this study had a lower risk of dementia than the lowest and highest intake groups, respectively. This was partly consistent with previous research on the association between moderate to higher intakes of magnesium or magnesium oxide and a lower risk of dementia [7,8,21]; our findings strengthened the evidence thanks to a large sample size. Another study found that the highest and lowest quintiles of plasma magnesium levels are associated with a subsequent higher risk of vascular dementia [22], a result consistent with the current findings.

One possible mechanism for the association between moderate magnesium intake and the lower risk of dementia is related to magnesium’s effect on neuronal excitability. N-methyl-D-aspartate receptors are permeable to calcium but can be blocked by sodium and magnesium ions to prevent the excitotoxicity induced by excessive neuronal activity [23,24]. We speculated that these relationships may be related to magnesium’s ability to suppress excessive neural activity; appropriate levels of neural activity are necessary for proper brain activity. Another possible mechanism is magnesium’s association with insulin resistance and diabetes. Magnesium supplementation improves the insulin resistance state [25] and a lower dietary intake of magnesium is linked to a greater risk of type 2 diabetes, which is robustly associated with a greater dementia risk [26]. Furthermore, a chronic magnesium deficiency increases the production of free radicals, which in turn increases the risk of a wide range of ageing-related diseases, including stroke and cardiovascular diseases [27]. Magnesium depletion increases the production of oxygen-derived free radicals, hydrogen peroxide, and superoxide anions by inflammatory cells [27]; aggravates oxygen stress; and weakens antioxidant defence [28,29]. Moreover, magnesium is required for the proper function of the γ-glutamine transpeptidase, which plays an important role in the synthesis of glutathione, an antioxidant; hence, magnesium may have a mild antioxidant effect [30,31]. However, whether adequate magnesium levels can prevent dementia must be investigated in future randomized clinical trials (RCTs).

The relationship between a higher intake of total sugars and a higher risk of dementia and the concomitant association of carbohydrates in this work was consistent with previous cohort studies and with the adverse effects of persistently higher blood sugar levels on the brain and nervous system. In the present study, a higher risk of dementia was found in the higher sugar intake groups than in the lower intake groups. This finding is consistent with previous reports stating that a higher risk of dementia is associated with a higher fructose intake [6] and a higher sugar intake from beverages [32]. In addition, animal studies revealed that the long-term consumption of sucrose-sweetened water causes insulin resistance, impairs memory function, and causes amyloid-β deposition [33], and a diet supplemented with liquid sucrose is associated with hippocampal inflammation and memory impairment [34]. In addition, diets high in fat, and sugar can reduce BDNF expression, which is associated with memory impairment [34]. In general, a higher sugar intake is linked to microvascular damage [35] and impaired glucose metabolism [36], which can damage the nervous system. Based on the above, a higher sugar intake may be associated with greater dementia risk. However, as certain dementia patients show sugar-preferring dietary patterns [37], future RCTs must focus on demonstrating a causal relationship, by implementing sugar restriction, for instance.

The link found in this study between the highest or lowest quintiles of fat intake and a higher risk of dementia was consistent with previous findings, which indicated that a higher fat intake is linked to an increased risk of cardiovascular disease and stroke and fat is an important nutrient for nerve cells. In the present work, a moderate fat intake was associated with a lower risk of dementia. This finding may be partly consistent with one study using a relatively smaller sample size than that of the present study (N = 937), which reported that a diet rich in protein and fat is associated with a lower subsequent risk of dementia [9]. A closer look at this relationship revealed that polyunsaturated fats may account for this association: saturated fats did not show a substantial association with dementia risk, whereas polyunsaturated fats showed a similar association with dementia risk. The lack of association found between saturated fats and increased dementia risk over time was not consistent with the findings of a middle-sized meta-analysis (8630 participants and 633 cases from four independent prospective cohort studies) [38]. The results obtained for polyunsaturated fat were not significant after multiple comparison correction, but a trend toward a lower risk of dementia was observed in the group with the second-highest polyunsaturated fat intake compared with that in the group with the lowest intake. This finding is consistent with previous studies showing that moderate fish intake or high unsaturated fat intake is associated with a lower risk of dementia [1,14]. In addition, a previous meta-analysis revealed a dose-dependent decline in the intake level of polyunsaturated fat correlated with mild cognitive impairment risk [39]. Moreover, fish consumption is associated with a lower risk of cardiovascular diseases [40]. Among polyunsaturated fats, docosahexaenoic and eicosapentaenoic acids are considered protective against neurodegeneration [41]. However, fish consumption can lead to an excessive intake of methylmercury [42], which has neurotoxic effects. Accordingly, a moderate fat intake may be associated with the lowest risk of dementia.

The association between moderate protein levels and a lower risk of dementia in this work is consistent with previous research and may be related to the importance of proteins in maintaining brain tissue integrity and the link of excessive meat intake to higher stroke and cardiovascular risk. One study using a sample size relatively smaller than that of the present study (N = 937) reported that a protein-rich diet is associated with a lower subsequent risk of dementia [9]. Similarly, our previous study using UK Biobank data showed that an overall moderate intake of meat and fish is linked to a lower risk of dementia [14]. Given that meat and fish are important protein sources, the present results are consistent with the above previous studies. Possible reasons why adequate protein levels are linked to a lower risk of dementia include the following: first, proteins are essential for the maintenance of neuronal membranes and neuronal integrity and second, certain amino acids are precursors of neurotransmitters [43]. Another possible mechanism is that proteins are important for muscle retention [43]. Further, a higher meat intake has been associated with an increased risk of cardiovascular diseases and stroke [43]. These findings may explain the observed association between moderate protein intake and the lower risk of dementia. However, these findings are only speculative and should be confirmed by future intervention and animal studies.

Although some results were not significant after correcting for multiple comparisons, their tendencies were consistent with previous studies. Additional research and RCT data are warranted for a more comprehensive understanding. For folate, the highest or lowest intake quintile was also linked to an increased risk of dementia. This finding was partly consistent with a meta-analysis linking a higher folate intake to a lower risk of dementia. However, previous results, even among meta-analyses, remain contradictory. For instance, another meta-analysis failed to find an association between higher folate intake and dementia [2]. Similarly, some meta-analyses found an association between higher vitamin E intake and a lower dementia risk [3] and others failed to find a connection [5]. The present results are consistent with the latter. Thus, the lack of cohesion between previous studies and the trends in the present work prevented us from drawing conclusions about the existence of a relationship. Although previous meta-analyses consistently found an association between vitamin D deficiency and greater dementia risk, we failed to find such a connection [1]. However, groups with intermediate vitamin D intake showed an uncorrected tendency to a lower risk of dementia risk over time. Perhaps, the present results may suggest the effect of an insufficient sample size. Further research is warranted to corroborate or disprove the existence of these relationships.

This study has several limitations. First, its prospective observational design. Although we corrected for a wide range of potentially confounding variables, the observed associations may have been influenced by the type of diet chosen by people at risk of developing dementia. In addition, this study excluded subjects who had developed dementia before two years after the online survey of the diet from the analysis. However, the neuropathological process underlying Alzheimer’s disease begins 20 years before the onset of the disease [44]. Therefore, specific eating behaviours may also be an expression of some preclinical behaviours. Moreover, there were a few years between the time when the subjects visited the facility for baseline measurements to when they completed the online survey on diet. Therefore, the time when the covariate data were measured and when the data on the variables of interest (i.e., diet) differed. These discrepancies suggested that our correction for covariates may not have been accurate. Finally, although the dietary data from the online diet survey are from 2009 to 2012, this study aimed to predict the onset of dementia up to 2021 but did not consider any changes in dietary habits after the survey. Although true for all subjects, this may have reduced the sensitivity of the statistical analysis.

This study investigated the relationship between the intake of 23 nutrients and the risk of dementia using modern data and a sufficient sample size after adjusting for a wide range of potential confounding factors. The main results were: (a) compared with no alcohol intake, any level of alcohol intake was associated with a lower risk of dementia; (b) compared with a higher protein intake, a moderate intake was associated with a lower risk of dementia; (c) compared with the highest or lowest quintiles of fat intake, a moderate intake was associated with a lower risk of dementia; the same trend was exhibited by polyunsaturated fatty acids (rather consistent with previous studies) but not by saturated fatty acids (not consistent with previous studies); (d) compared with a lower sugar intake, a higher total intake was associated with a higher risk of dementia. The same trend was found for carbohydrates with sugar components; (e) compared with the highest and lowest magnesium intakes, a moderately higher intake was associated with a lower risk of dementia; (f) other than folic acid, which showed a certain association with dementia, the overall association between vitamin intake and dementia described in previous studies could not be replicated; (g) we found no trend of associations between nutrients such as calcium, retinol, etc, and dementia. Points (b), (c), (e–g) are findings that had not been previously established. The association between nutrients and dementia risk was previously reported in studies with relatively smaller sample sizes than that of the present study. However, the unprecedentedly large sample size and the correction for confounding factors are important points of the present study. Overall, the present findings are congruent with the importance of a moderate intake of certain nutrients.

## Figures and Tables

**Table 1:** Baseline characteristics of participants with and without incident dementia.

	No Incident Dementia (*n* = 160,170)	Incident Dementia (*n* = 1206)
		Mean
Age	58.51 (8.01)	66.27 (5.19)
Townsend deprivation index	−1.62 (2.84)	−1.48 (2.94)
Education length	15.49 (4.79)	14.3 (5.07)
MET *	30.27 (32.04)	32.1 (36.08)
Height	169.66 (9.17)	169.48 (9.13)
Depression score	5.42 (1.87)	5.47 (1.94)
Visuospatial memory (errors)	3.5 (2.36)	3.93 (2.5)
		Number
Male number	74,596 (46.6%)	699 (58%)
Household income		
(a) Less than £18,000	22,864 (14.3%)	359 (29.8%)
(b) £18,000 to £30,999	37,766 (23.6%)	404 (33.5%)
(c) £31,000 to £5,1999	46,066 (28.8%)	266 (22.1%)
(d) £52,000 to £100,000	41,033 (25.6%)	140 (11.6%)
(e) Greater than £100,000	12,441 (7.8%)	37 (3.1%)
Currently employed	102,800 (64.2%)	337 (27.9%)
BMI		
Underweight (x ≤ 18.5)	819 (0.5%)	10 (0.8%)
Normal (18.5 < x ≤ 25)	59,474 (37.1%)	409 (33.9%)
Overweight (25 < x ≤ 30)	66,943 (41.8%)	476 (39.5%)
Obesity (x < 30)	32,934 (20.6%)	311 (25.8%)
Household number		
(a) 1	28,380 (17.7%)	281 (23.3%)
(b) 2	73,416 (45.8%)	754 (62.5%)
(c) 3	25,180 (15.7%)	101 (8.4%)
(d) 4≤	33,194 (20.7%)	70 (5.8%)
Overall health (4 levels)		
(a) Poor	4326 (2.7%)	88 (7.3%)
(b) Fair	26,009 (16.2%)	287 (23.8%)
(c) Good	95,771 (59.8%)	673 (55.8%)
(d) Excellent	34,064 (21.3%)	158 (13.1%)
Sleep duration		
(a) ≤4 h,	911 (0.6%)	11 (0.9%)
(b) 5 h or 6 h,	34,352 (21.4%)	266 (22.1%)
(c) 7 h or 8 h,	114,998 (71.8%)	816 (67.7%)
(d) 9 h≤	9909 (6.2%)	113 (9.4%)
Current smoking level (3 levels)		
(a) No	147,642 (92.2%)	1111 (92.1%)
(b) Only occasionally	4021 (2.5%)	29 (2.4%)
(c) On most or all days	8507 (5.3%)	66 (5.5%)
Diastolic BP		
x < 65	5626 (3.5%)	47 (3.9%)
65 ≤ x < 90	121,089 (75.6%)	918 (76.1%)
90 ≤ 30	33,455 (20.9%)	241 (20%)
Ethnicity (non-white)	5631 (3.5%)	19 (1.6%)
Antihypertensive drug intake	27,627 (17.2%)	431 (35.7%)
Statin intake	15,590 (9.7%)	239 (19.8%)
Diabetes	6159 (3.8%)	120 (10%)
Heart attack	2535 (1.6%)	59 (4.9%)
Angina	3270 (2%)	95 (7.9%)
Stroke	1589 (1%)	52 (4.3%)
Cancer	12,781 (8%)	128 (10.6%)
Other serious medical conditions	31,084 (19.4%)	408 (33.8%)

* MET: metabolic equivalent of task hours (MET). Physical activity level.

**Table 2:** Nutrient intake level (amount, adjusted HR, and case ratio) for each group and uncorrected and corrected *p* values of overall group differences.

Nutrients	Amount (Upper), Adjusted HR (Middle), Case Number/Entire Sample (%)					*p* (FDR)
(Unit)	Level 1	Level 2	Level 3	Level 4	Level 5	
alcohol	x ≤ 0	0 < x ≤ 3.2533	3.2533 < x ≤14.74	14.74 < x ≤ 30.45	30.45 < x	
(g)	reference	0.79 (0.62,1.02)	0.85 (0.72,1)	0.77 (0.65,0.9)	0.79 (0.67,0.93)	0.026
	462/52,407 (0.9%)	71/9352 (0.8%)	226/32,299 (0.7%)	215/33,446 (0.6%)	232/33,872 (0.7%)	
calcium	x ≤ 688.02	688.02 < x ≤ 853.05	853.05 < x ≤ 1011.97	1011.97 < x ≤ 1226.67	1226.67 < x	
(mg)	reference	1.11 (0.92,1.34)	1.07 (0.89,1.29)	1.01 (0.84,1.23)	1.21 (1.01,1.45)	0.324
	200/31,483 (0.6%)	237/32,560 (0.7%)	241/32,671 (0.7%)	239/32,663 (0.7%)	289/31,999 (0.9%)	
carbohydrate	x ≤ 187.33	187.33 < x ≤ 227.05	227.05 < x ≤ 263.84	263.84 < x ≤ 312.90	312.90 < x	
(g)	reference	0.86 (0.71,1.04)	0.91 (0.76,1.1)	1.07 (0.89,1.28)	1.18 (0.98,1.4)	0.026
	225/31,682 (0.7%)	200/32,274 (0.6%)	218/32,544 (0.7%)	265/32,813 (0.8%)	298/32,063 (0.9%)	
carotene	x ≤ 991.92	991.92 < x ≤ 1996.76	1996.76 < x ≤ 3107.49	3107.49 < x ≤ 4748.76	4748.76 < x	
(µg)	reference	0.93 (0.78,1.12)	0.9 (0.75,1.08)	0.91 (0.76,1.1)	1.07 (0.9,1.27)	0.328
	240/31,192 (0.8%)	225/32,817 (0.7%)	225/32,932 (0.7%)	231/32,641 (0.7%)	285/31,794 (0.9%)	
energy	x ≤ 6757.23	6757.23 < x ≤ 7976.07	7976.07 < x ≤ 9124.26	9124.26 < x ≤ 10,674.42	10,674.42 < x	
(KJ)	reference	1.01 (0.84,1.21)	0.96 (0.8,1.16)	0.89 (0.73,1.07)	1.15 (0.96,1.38)	0.126
	224/30,924 (0.7%)	235/32,216 (0.7%)	236/32,667 (0.7%)	221/33,035 (0.7%)	290/32,534 (0.9%)	
Englyst dietary fibre	x ≤ 11.10	11.10 < x ≤ 14.22	14.22 < x ≤ 17.19	17.19 < x ≤ 21.14	21.14 < x	
(g)	reference	0.94 (0.78,1.13)	0.83 (0.69,1)	0.98 (0.82,1.18)	0.99 (0.83,1.18)	0.336
	224/31,504 (0.7%)	227/32,513 (0.7%)	209/32,632 (0.6%)	263/32,718 (0.8%)	283/32,009 (0.9%)	
fat	x ≤ 52.97	52.97 < x ≤ 67.44	67.44 < x ≤ 81.38	81.38 < x ≤ 100.03	100.03 < x	
(g)	reference	0.8 (0.67,0.95)	0.83 (0.69,0.99)	0.76 (0.64,0.91)	1 (0.84,1.18)	0.025
	266/31,328 (0.8%)	219/32,136 (0.7%)	232/32,681 (0.7%)	213/32,695 (0.7%)	276/32,536 (0.8%)	
folate	x ≤ 213.92	213.92 < x ≤ 265.13	265.13 < x ≤ 314.08	314.08 < x ≤ 381.53	381.53 < x	
(µg)	reference	0.84 (0.69,1.01)	0.8 (0.66,0.96)	0.88 (0.74,1.06)	0.98 (0.82,1.17)	0.126
	231/31,317 (0.7%)	216/32,488 (0.7%)	213/32,643 (0.7%)	250/32,654 (0.8%)	296/32,274 (0.9%)	
food weight	x ≤ 2552.33	2552.33 < x ≤ 2940.00	2940.00 < x ≤ 3302.85	3302.85 < x ≤ 3782.00	3782.00 < x	
(g)	reference	0.95 (0.79,1.14)	0.89 (0.74,1.07)	0.93 (0.78,1.12)	1.07 (0.9,1.28)	0.35
	247/31,082 (0.8%)	237/32,243 (0.7%)	225/32,558 (0.7%)	233/32,805 (0.7%)	264/32,688 (0.8%)	
iron	x ≤ 10.08	10.08 < x ≤ 12.30	12.30 < x ≤ 14.34	14.34 < x ≤ 16.96	16.96 < x	
(mg)	reference	0.92 (0.77,1.11)	0.85 (0.7,1.02)	0.83 (0.69,1)	1.02 (0.86,1.22)	0.132
	231/30,826 (0.7%)	228/32,230 (0.7%)	219/32,464 (0.7%)	229/33,002 (0.7%)	299/32,854 (0.9%)	
magnesium	x ≤ 263.40	263.40 < x ≤ 313.92	313.92 < x ≤ 360.46	360.46 < x ≤ 422.92	422.92 < x	
(mg)	reference	0.98 (0.81,1.18)	0.91 (0.76,1.1)	0.81 (0.67,0.98)	1.14 (0.95,1.36)	0.025
	225/30,903 (0.7%)	237/32,185 (0.7%)	228/32,590 (0.7%)	213/32,937 (0.6%)	303/32,761 (0.9%)	
polyunsaturated fat	x ≤ 8.32	8.32 < x ≤ 11.55	11.55 < x ≤ 14.91	14.91 < x ≤ 19.51	19.51 < x	
(g)	reference	0.87 (0.73,1.04)	0.89 (0.75,1.06)	0.74 (0.61,0.89)	0.89 (0.75,1.06)	0.078
	275/31,462 (0.9%)	242/32,390 (0.7%)	244/32,726 (0.7%)	202/32,515 (0.6%)	243/32,283 (0.8%)	
potassium	x ≤ 2821.42	2821.42 < x ≤ 3377.58	3377.58 < x ≤ 3893.73	3893.73 < x ≤ 4571.70	4571.70 < x	
(mg)	reference	0.86 (0.71,1.04)	0.92 (0.76,1.1)	0.87 (0.73,1.05)	1.05 (0.88,1.26)	0.18
	222/31,075 (0.7%)	210/32,439 (0.6%)	233/32,666 (0.7%)	238/32,866 (0.7%)	303/32,330 (0.9%)	
protein	x ≤ 62.51	62.51 < x ≤ 74.76	74.76 < x ≤ 85.60	85.60 < x ≤ 99.95	99.95 < x	
(g)	reference	0.98 (0.82,1.18)	0.94 (0.78,1.13)	0.87 (0.72,1.05)	1.17 (0.98,1.4)	0.044
	226/31,191 (0.7%)	238/32,504 (0.7%)	230/32,577 (0.7%)	219/32,710 (0.7%)	293/32,394 (0.9%)	
retinol	x ≤ 176.18	176.18 < x ≤ 259.66	259.66 < x ≤ 345.04	345.04 < x ≤ 459.58	459.58 < x	
(µg)	reference	0.96 (0.8,1.16)	0.94 (0.78,1.14)	1 (0.83,1.2)	0.97 (0.81,1.17)	0.814
	221/31,216 (0.7%)	224/31,751 (0.7%)	228/31,963 (0.7%)	251/32,225 (0.8%)	259/32,048 (0.8%)	
saturated fat	x ≤ 19.28	19.28 < x ≤ 25.25	25.25 < x ≤ 31.14	31.14 < x ≤ 39.24	39.24 < x	
(g)	reference	0.9 (0.75,1.08)	0.95 (0.79,1.14)	1.04 (0.87,1.24)	1.06 (0.89,1.27)	0.393
	232/31,383 (0.7%)	218/32,302 (0.7%)	230/32,601 (0.7%)	257/32,611 (0.8%)	269/32,479 (0.8%)	
starch	x ≤ 84.78	84.78 < x ≤ 108.32	108.32 < x ≤ 129.57	129.57 < x ≤ 156.59	156.59 < x	
(g)	reference	0.93 (0.78,1.11)	0.97 (0.81,1.16)	0.82 (0.68,0.99)	0.97 (0.81,1.16)	0.328
	244/31,383 (0.8%)	240/32,122 (0.7%)	249/32,585 (0.8%)	216/32,807 (0.7%)	257/32,479 (0.8%)	
total sugars	x ≤ 80.88	80.88 < x ≤ 103.52	103.52 < x ≤ 125.50	125.50 < x ≤ 155.11	155.11 < x	
(g)	reference	0.82 (0.68,1)	0.92 (0.76,1.11)	1.07 (0.89,1.28)	1.27 (1.07,1.51)	0.001
	220/31,923 (0.7%)	186/32,566 (0.6%)	214/32,579 (0.7%)	261/32,558 (0.8%)	325/31,750 (1%)	
vitamin B6	x ≤ 1.59	1.59 < x ≤ 1.95	1.95 < x ≤ 2.29	2.29 < x ≤ 2.72	2.72 < x	
(mg)	reference	0.94 (0.77,1.13)	0.97 (0.8,1.16)	0.94 (0.78,1.13)	1.1 (0.92,1.31)	0.369
	213/31,540 (0.7%)	217/32,686 (0.7%)	235/32,843 (0.7%)	242/32,350 (0.7%)	299/31,957 (0.9%)	
vitamin B12	x ≤ 3.18	3.18 < x ≤ 4.65	4.65 < x ≤ 6.31	6.31 < x ≤ 9.07	9.07 < x	
(µg)	reference	1.05 (0.87,1.26)	1 (0.83,1.2)	0.99 (0.83,1.19)	0.98 (0.82,1.18)	0.814
	216/31,639 (0.7%)	247/32,359 (0.8%)	242/32,335 (0.7%)	244/32,617 (0.7%)	257/32,426 (0.8%)	
vitamin C	x ≤ 69.00	69.00 < x ≤ 109.51	109.51 < x ≤ 154.23	154.23 < x ≤ 217.06	217.06 < x	
(mg)	reference	0.97 (0.81,1.17)	1 (0.83,1.21)	1.01 (0.84,1.22)	1.12 (0.93,1.34)	0.549
	221/31,367 (0.7%)	235/32,293 (0.7%)	245/32,419 (0.8%)	246/32,809 (0.7%)	259/32,488 (0.8%)	
vitamin D	x ≤ 0.97	0.97 < x ≤ 1.65	1.65 < x ≤ 2.52	2.52 < x ≤ 4.23	4.23 < x	
(µg)	reference	0.91 (0.76,1.1)	0.87 (0.73,1.04)	0.83 (0.7,1)	0.96 (0.81,1.14)	0.344
	238/31,738 (0.7%)	234/32,610 (0.7%)	232/32,269 (0.7%)	226/32,382 (0.7%)	276/32,377 (0.9%)	
vitamin E	x ≤ 5.73	5.73 < x ≤ 7.64	7.64 < x ≤ 9.54	9.54 < x ≤ 12.19	12.19 < x	
(mg)	reference	0.98 (0.82,1.17)	1 (0.84,1.2)	1.02 (0.85,1.22)	1.02 (0.85,1.22)	0.814
	241/31,182 (0.8%)	235/32,358 (0.7%)	242/32,719 (0.7%)	244/32,728 (0.7%)	244/32,389 (0.8%)	

**Table 3:** Nutrient intake level (amount and adjusted HR as well as 95% CI) of each group and corrected *p* values of overall group differences after adjusting for the quintiles of energy intake level (left) and P values in the main analyses (right).

Nutrients	Amount (Upper), Adjusted HR (Lower)					*p* (Group, FDR)
(Unit)	Level 1	Level 2	Level 3	Level 4	Level 5	
alcohol	x ≤ 0	0 < x ≤ 3.2533	3.2533 < x ≤ 14.74	14.74 < x ≤ 30.45	30.45 < x	0.023
(g)	reference	0.79 (0.61,1.02): 0.79 (0.62,1.02)	0.85 (0.72,1): 0.85 (0.72,1)	0.76 (0.65,0.9): 0.77 (0.65,0.9)	0.77 (0.65,0.91): 0.79 (0.67,0.93)	
calcium	x ≤ 688.02	688.02 < x ≤ 853.05	853.05 < x ≤ 1011.97	1011.97 < x ≤ 1226.67	1226.67 < x	0.516
(mg)	reference	1.13 (0.93,1.37): 1.11 (0.92,1.34)	1.1 (0.9,1.34): 1.07 (0.89,1.29)	1.04 (0.84,1.28): 1.01 (0.84,1.23)	1.19 (0.96,1.49): 1.21 (1.01,1.45)	
carbohydrate	x ≤ 187.33	187.33 < x ≤ 227.05	227.05 < x ≤ 263.84	263.84 < x ≤ 312.90	312.90 < x	0.023
(g)	reference	0.9 (0.73,1.11): 0.86 (0.71,1.04)	1.03 (0.82,1.29): 0.91 (0.76,1.1)	1.31 (1.03,1.68): 1.07 (0.89,1.28)	1.44 (1.09,1.91): 1.18 (0.98,1.4)	
carotene	x ≤ 991.92	991.92 < x ≤ 1996.76	1996.76 < x ≤ 3107.49	3107.49 < x ≤ 4748.76	4748.76 < x	0.440
(ug)	reference	0.93 (0.77,1.12): 0.93 (0.78,1.12)	0.89 (0.74,1.08): 0.9 (0.75,1.08)	0.91 (0.75,1.09): 0.91 (0.76,1.1)	1.05 (0.88,1.25): 1.07 (0.9,1.27)	
Englyst diet fiber	x ≤ 11.10	11.10 < x ≤ 14.22	14.22 < x ≤ 17.19	17.19 < x ≤ 21.14	21.14 < x	0.440
(g)	reference	0.94 (0.78,1.13): 0.94 (0.78,1.13)	0.82 (0.68,1): 0.83 (0.69,1)	0.97 (0.8,1.17): 0.98 (0.82,1.18)	0.94 (0.77,1.15): 0.99 (0.83,1.18)	
fat	x ≤ 52.97	52.97 < x ≤ 67.44	67.44 < x ≤ 81.38	81.38 < x ≤ 100.03	100.03 < x	0.032
(g)	reference	0.75 (0.61,0.91): 0.8 (0.67,0.95)	0.76 (0.61,0.94): 0.83 (0.69,0.99)	0.68 (0.53,0.87): 0.76 (0.64,0.91)	0.81 (0.62,1.07): 1 (0.84,1.18)	
folate	x ≤ 213.92	213.92 < x ≤ 265.13	265.13 < x ≤ 314.08	314.08 < x ≤ 381.53	381.53 < x	0.248
(µg)	reference	0.83 (0.69,1.01): 0.84 (0.69,1.01)	0.79 (0.65,0.96): 0.8 (0.66,0.96)	0.87 (0.71,1.05): 0.88 (0.74,1.06)	0.93 (0.76,1.14): 0.98 (0.82,1.17)	
food weight	x ≤ 2552.33	2552.33 < x ≤ 2940.00	2940.00 < x ≤ 3302.85	3302.85 < x ≤ 3782.00	3782.00 < x	0.614
(g)	reference	0.95 (0.79,1.14): 0.95 (0.79,1.14)	0.89 (0.74,1.08): 0.89 (0.74,1.07)	0.92 (0.76,1.12): 0.93 (0.78,1.12)	1.02 (0.84,1.25): 1.07 (0.9,1.28)	
iron	x ≤ 10.08	10.08 < x ≤ 12.30	12.30 < x ≤ 14.34	14.34 < x ≤ 16.96	16.96 < x	0.328
(mg)	reference	0.9 (0.75,1.1): 0.92 (0.77,1.11)	0.82 (0.67,1.01): 0.85 (0.7,1.02)	0.8 (0.64,1): 0.83 (0.69,1)	0.93 (0.74,1.17): 1.02 (0.86,1.22)	
magnesium	x ≤ 263.40	263.40 < x ≤ 313.92	313.92 < x ≤ 360.46	360.46 < x ≤ 422.92	422.92 < x	0.111
(mg)	reference	0.97 (0.8,1.18): 0.98 (0.81,1.18)	0.9 (0.73,1.12): 0.91 (0.76,1.1)	0.8 (0.64,1.01): 0.81 (0.67,0.98)	1.07 (0.84,1.37): 1.14 (0.95,1.36)	
polyunsaturated fat	x ≤ 8.32	8.32 < x ≤ 11.55	11.55 < x ≤ 14.91	14.91 < x ≤ 19.51	19.51 < x	0.023
(g)	reference	0.84 (0.7,1.01): 0.87 (0.73,1.04)	0.84 (0.7,1.02): 0.89 (0.75,1.06)	0.67 (0.55,0.83): 0.74 (0.61,0.89)	0.75 (0.6,0.93): 0.89 (0.75,1.06)	
potassium	x ≤ 2821.42	2821.42 < x ≤ 3377.58	3377.58 < x ≤ 3893.73	3893.73 < x ≤ 4571.70	4571.70 < x	0.440
(mg)	reference	0.86 (0.71,1.05): 0.86 (0.71,1.04)	0.92 (0.75,1.13): 0.92 (0.76,1.1)	0.87 (0.7,1.09): 0.87 (0.73,1.05)	1.01 (0.8,1.27): 1.05 (0.88,1.26)	
protein	x ≤ 62.51	62.51 < x ≤ 74.76	74.76 < x ≤ 85.60	85.60 < x ≤ 99.95	99.95 < x	0.248
(g)	reference	0.99 (0.82,1.2): 0.98 (0.82,1.18)	0.95 (0.78,1.17): 0.94 (0.78,1.13)	0.89 (0.71,1.11): 0.87 (0.72,1.05)	1.15 (0.91,1.45): 1.17 (0.98,1.4)	
retinol	X ≤ 176.18	176.18 < x ≤ 259.66	259.66 < x ≤ 345.04	345.04 < x ≤ 459.58	459.58 < x	0.790
(µg)	reference	0.96 (0.8,1.16): 0.96 (0.8,1.16)	0.94 (0.77,1.14): 0.94 (0.78,1.14)	0.99 (0.81,1.2): 1 (0.83,1.2)	0.92 (0.75,1.13): 0.97 (0.81,1.17)	
saturated fat	X ≤ 19.28	19.28 < x ≤ 25.25	25.25 < x ≤ 31.14	31.14 < x ≤ 39.24	39.24 < x	0.629
(g)	reference	0.92 (0.76,1.12): 0.9 (0.75,1.08)	0.99 (0.8,1.21): 0.95 (0.79,1.14)	1.08 (0.87,1.35): 1.04 (0.87,1.24)	1.03 (0.81,1.32): 1.06 (0.89,1.27)	
starch	X ≤ 84.78	84.78 < x ≤ 108.32	108.32 < x ≤ 129.57	129.57 < x ≤ 156.59	156.59 < x	0.328
(g)	reference	0.91 (0.76,1.1): 0.93 (0.78,1.11)	0.94 (0.77,1.14): 0.97 (0.81,1.16)	0.78 (0.63,0.96): 0.82 (0.68,0.99)	0.85 (0.68,1.06): 0.97 (0.81,1.16)	
total sugars	X ≤ 80.88	80.88 < x ≤ 103.52	103.52 < x ≤ 125.50	125.50 < x ≤ 155.11	155.11 < x	0.001
(g)	reference	0.85 (0.7,1.04): 0.82 (0.68,1)	0.98 (0.8,1.19): 0.92 (0.76,1.11)	1.17 (0.96,1.43): 1.07 (0.89,1.28)	1.4 (1.13,1.73): 1.27 (1.07,1.51)	
vitamin B6	X ≤ 3.18	3.18 < x ≤ 4.65	4.65 < x ≤ 6.31	6.31 < x ≤ 9.07	9.07 < x	0.666
(mg)	reference	0.94 (0.78,1.15): 0.94 (0.77,1.13)	0.98 (0.8,1.19): 0.97 (0.8,1.16)	0.95 (0.78,1.16): 0.94 (0.78,1.13)	1.07 (0.87,1.31): 1.1 (0.92,1.31)	
vitamin B12	X ≤ 1.59	1.59 < x ≤ 1.95	1.95 < x ≤ 2.29	2.29 < x ≤ 2.72	2.72 < x	0.790
(µg)	reference	1.04 (0.87,1.26): 1.05 (0.87,1.26)	0.99 (0.82,1.2): 1 (0.83,1.2)	0.98 (0.81,1.18): 0.99 (0.83,1.19)	0.96 (0.8,1.16): 0.98 (0.82,1.18)	
vitamin C	X ≤ 69.00	69.00 < x ≤ 109.51	109.51 < x ≤ 154.23	154.23 < x ≤ 217.06	217.06 < x	0.666
(mg)	reference	0.97 (0.81,1.17): 0.97 (0.81,1.17)	1 (0.83,1.21): 1 (0.83,1.21)	1.01 (0.84,1.22): 1.01 (0.84,1.22)	1.1 (0.91,1.33): 1.12 (0.93,1.34)	
vitamin D	X ≤ 0.97	0.97 < x ≤ 1.65	1.65 < x ≤ 2.52	2.52 < x ≤ 4.23	4.23 < x	0.398
(µg)	reference	0.91 (0.76,1.09): 0.91 (0.76,1.1)	0.86 (0.71,1.03): 0.87 (0.73,1.04)	0.82 (0.68,0.99): 0.83 (0.7,1)	0.94 (0.78,1.12): 0.96 (0.81,1.14)	
Vitamin E	X ≤ 5.73	5.73 < x ≤ 7.64	7.64 < x ≤ 9.54	9.54 < x ≤ 12.19	12.19 < x	0.800
(mg)	reference	0.98 (0.81,1.18): 0.98 (0.82,1.17)	1 (0.82,1.21): 1 (0.84,1.2)	1 (0.82,1.23): 1.02 (0.85,1.22)	0.95 (0.76,1.18): 1.02 (0.85,1.22)	

## Data Availability

Researchers can apply to use the UK Biobank resource (https://www.ukbiobank.ac.uk/ (accessed on 9 September 2022)) and access the data used in this study.

## Supplementary Materials

The following supporting information can be downloaded at: https://www.mdpi.com/article/10.3390/nu15040842/s1, Table S1: Characteristics of the subjects who lack ≥1 covariates and those who have complete covariate data among the participants in the UK Biobank not excluded according to the four excluding criteria of dementia who completed the online survey and had nutritional data; Method S1: Supplemental Methods Supplemental Table S1. Characteristics of the subjects who lack ≥1 covariates and those who have complete covariate data among the participants in the UK Biobank not excluded according to the four excluding criteria of dementia who completed the online survey and had nutritional data [14,17,45,46,47,48,49,50,51,52].
