# Supplementary material for: Nutrients and Dementia: Prospective Study
Source: Nutrients. 2023 Feb 7;15(4):842. doi: 10.3390/nu15040842 (PMC9960559; doi:10.3390/nu15040842)
Supplement: Supplementary file 1 [file nutrients-15-00842-s001.zip › nutrients-2062229-supplementary.pdf]

Supplementary online materials

Supplemental methods

Additional information on study subjects

Adults 40–69 years old registered with the National Health Service (NHS) who lived within 25 miles of the evaluation sites were invited by email to participate in the UK Biobank project. No exclusion criteria were applied for recruitment. The descriptions in this subsection were largely reproduced from another study that used UK Biobank data (Takeuchi and Kawashima, 2021).

Details on sociodemographic and lifestyle measures

The descriptions in this subsection were largely reproduced from another study that used UK Biobank data (Takeuchi and Kawashima, 2021).

Cov1: Neighborhood-level socioeconomic status was measured using the Townsend index of material deprivation (Townsend, 1987) (via UK Biobank Field ID: 189). Status was based on the postcode of the subject's address and represented a composite index of four postcode-level socioeconomic status variables: household overcrowding, unemployment, nonhome ownership, and non-car ownership. A high score implies a low socioeconomic status. For this variable, only the value at recruitment was available,

which was used for all analyses.

Cov2: Education level was based on self-reported data (via UK Biobank Field ID: 6138). Education level categories were transformed into the following numerical values (Okbay et al., 2016): “College or University degree” = 20 years; “levels/AS levels or equivalent” = 13 years; “O levels/GCSEs or equivalent” = 10 years; “CSEs or equivalent” = 10 years; “NVQ or HND or HNC or equivalent“ = 19 years; “Other professional qualifications, e.g., nursing, teaching” = 15 years; “None of the above” = 7 years; and “Prefer not to answer” = missing. For this variable, only the value at recruitment was available, which was used for all analyses.

Cov3: Household income was the self-reported total income (before taxes) of the subject’s household (via UK Biobank Field ID: 738). The available choices were <£18,000, £18,000 to £30,999, £100,000, £31,000 to £51,999, £52,000 to £100,000, >£100,000, “do not know,” and “prefer not to answer.” These choices were converted into ordinal variables between 1 and 5 (>£100,000 = 5) (Shen et al., 2018) after excluding the answers “do not know” and “prefer not to answer”.

Cov4: “Current employment status” was used to describe the participants’ occupations (UK Biobank Field ID: 6142). The responses to the variable were “In paid employment or self-employed,” “Retired,” “Looking after home and/or family,” “Unable to work

because of sickness or disability," "Unemployed," "Doing unpaid or voluntary work," "Full or part-time student," and "None of the above." Multiple responses were allowed. Subject responses were classified as either "In paid employment or self-employed" or not.

Cov5: The physical activity level was calculated from the recorded items i the International Physical Activity Questionnaire short form and converted into a single measure of total physical activity in the metabolic equivalent of task hours (data-field IDs: 864, 874, 884, 894, 904, and 914). For additional details, see (Cullen et al., 2018).

Cov6: Participants were asked about the number of people in their household (including institutions such as care homes) (data-field IDs: 709). Answers were assigned to one of the following four variables: 1 (single person), 2 (two people), 3 (three people), and 4 (≥4 people) (Sarkar et al., 2008).

Cov7 and Cov8: Body weight was measured using Tanita BC418MA scales (data-field IDs: 21002). Height was determine using a Seca height measure (data-field IDs: 50). BMI was calculated from the measured height and weight. For the analyses, BMI was treated as a categorical variable and categorized as 30>x (obesity), 30≥x>25 (overweight), 25≥x>18.5 (normal), and 18.5≥x (underweight).

Cov9: Participants were asked about their health status. Possible answers were excellent,

good, fair, and poor, which were respectively converted to values of 4, 3, 2, and 1, prior to input in the statistical analyses (data-field IDs: 2178).

Cov10: Sleep duration was assessed with the item "About how many hours sleep do you get every 24 h? (Please include naps.)" (data-field IDs: 1160). Responses were coded as integers and divided into four groups, (a) ≤4 h, (b) 5 or 6 h, (c) 7 or 8 h, and (d) ≤9 h.

Cov11: Diastolic blood pressure (BP) was measured using a digital BP monitor (Omron) or a manual sphygmomanometer when the digital monitor was unavailable (data-field IDs: 94, 4079). After taking 1–2 readings, they were averaged (Veldsman et al., 2020). For the analyses, diastolic BP was treated as a categorical variable and split into three groups (a) 65>x, (b) 90>x≥65, and (c) x≥90.

Cov12: Participants were asked about their current tobacco smoking status (data-field ID: 1239). Possible answers were 1 (No), 2 (Only occasionally), and 3 (Yes, on most or all days). Current tobacco smoking status was treated as a categorical variable. Responses of "prefer not to answer" were excluded.

Cov13: Ethnicity was self-reported; possible effective answers were divided into white or other and analyzed (data-field ID: 21000).

Cov14–Cov19: Participants were asked about the existence of a medical diagnosis of diabetes, heart attack, angina, stroke, cancer, and other serious medical conditions (item

IDs: 1049, 6150, 2453, and 2473). A dichotomized variable for the existence of each condition was generated based on this answer.

Cov20: Visuospatial memory was measured using the “pairs-matching” task. Participants were asked to memorize the positions of six card pairs and match these cards from memory with as few errors as possible. Scores on the pair-matching test corresponded to the number of errors that participants; high scores reflect poor cognitive functions (data-field ID: 399).

Cov21: Depressive symptoms were measured using the four-item Patient Health Questionnaire-4 (PHQ-4) (Batty et al., 2016), which was administered at all four patients visits to assessment centers (data-field IDs: 2050, 2060, 2070, and 2080). This measurement has an area under the curve of 0.79 for its correlation with a depression diagnosis (Khubchandani et al., 2016). For other information on the reliability and validity of this measurement see (Khubchandani et al., 2016).

Cov22: Patients who reported taking a BP medication (via UK Biobank Field IDs 6153) were coded as 1; others were coded as 0.

Cov23: Data on a history of statin use were collected using a touchscreen questionnaire at each assessment visit. A dichotomized variable (subjects who reported the use and those who did not) was generated based on this answer.

Determination of dementia

Previous methods (Lourida et al., 2019) were adopted to determine all-cause dementia. The descriptions in this subsection are largely reproduced from another study that used UK Biobank data (Takeuchi and Kawashima, 2021). All-cause dementia was determined based on hospital inpatient records containing data on admissions and diagnoses from the Hospital Episode Statistics for England, Scottish Morbidity Record Data for Scotland, and the Patient Episode Database for Wales. Additional cases were identified through the death register data provided by the NHS Digital for England and Wales and the Information and Statistics Division for Scotland. Diagnoses were recorded using the International Classification of Diseases (ICD) coding system. Participants with dementia were identified as having a primary/secondary diagnosis (hospital records) or underlying/contributory cause of death (death register) using the ICD-9 and ICD-10 codes for Alzheimer's disease and other dementia classifications.

Supplemental Table S1. Characteristics of the subjects who lack ≥1 covariates and those who have complete covariate data among the participants in the UK Biobank not excluded according to the four excluding criteria of dementia who completed the online survey and had nutritional data

	Excluded	Included	Cohen's d
Age	59.81(39982)	58.55(161351)	0.157
Townsend deprivation index	-1.4(39731)	-1.62(161351)	0.078
Education length	13.88(39085)	15.48(161351)	0.329
Household income	2.47(19232)	2.89(161351)	0.352
MET[1]	29.41(35670)	30.28(161351)	0.027
Standing height	167.23(39599)	169.66(161351)	0.264
Depression score	5.59(24953)	5.42(161351)	0.093
Visuospatial memory (errors)	3.62(39982)	3.5(161351)	0.052
BMI[2] (raw value)	27.21(39444)	26.9(161351)	0.066
Household number (4 levels:1,2,3,4≤)	2.36(39209)	2.39(161351)	0.036
Current smoking level (3 levels)	1.14(39905)	1.13(161351)	0.018
Sleep duration (hours, raw values)	7.12(39390)	7.17(161351)	0.041
Diastolic BP[3] (raw value)	81.93(39828)	81.78(161351)	0.014
			Odds ratio
Male	36.32%(39982)	46.66%(161351)	0.652
Currently employed	51.14%(39508)	63.91%(161351)	0.591
Non-white	106.53%(39332)	103.5%(161351)	1.927
Antihypertensive drug intake	20.74%(39148)	17.39%(161351)	1.243
Statin use	11.2%(39982)	9.81%(161351)	1.159
Diabetes	5.17%(39649)	3.89%(161351)	1.346
Heart attack	1.84%(39734)	1.61%(161351)	1.145
Angina	2.95%(39734)	2.09%(161351)	1.426
Stroke	1.3%(39734)	1.02%(161351)	1.286
Cancer	9.02%(39952)	8%(161351)	1.14
Other serious medical conditions	21.47%(36900)	19.51%(161351)	1.128

[1]MET:metabolic equivalent of task hours (MET). Physical activity level. [2]BMI:body mass index (BMI). [3]BP:blood pressure (BP).

References

Batty, G.D., Mcintosh, A.M., Russ, T.C., Deary, I.J., and Gale, C.R. (2016). Psychological distress, neuroticism, and cause-specific mortality: early prospective evidence from UK Biobank. *J Epidemiol Community Health* 70**,** 1136-1139.

Cullen, B., Newby, D., Lee, D., Lyall, D.M., Nevado-Holgado, A.J., Evans, J.J., Pell, J.P., Lovestone, S., and Cavanagh, J. (2018). Cross-sectional and longitudinal analyses of outdoor air pollution exposure and cognitive function in UK Biobank. *Scientific reports* 8**,** 1-14.

Khubchandani, J., Brey, R., Kotecki, J., Kleinfelder, J., and Anderson, J. (2016). The psychometric properties of PHQ-4 depression and anxiety screening scale among college students. *Archives of psychiatric nursing* 30**,** 457-462.

Lourida, I., Hannon, E., Littlejohns, T.J., Langa, K.M., Hyppönen, E., Kuźma, E., and Llewellyn, D.J. (2019). Association of lifestyle and genetic risk with incidence of dementia. *Jama* 322**,** 430-437.

Okbay, A., Beauchamp, J.P., Fontana, M.A., Lee, J.J., Pers, T.H., Rietveld, C.A., Turley, P., Chen, G.B., Emilsson, V., Meddens, S.F., Oskarsson, S., Pickrell, J.K., Thom, K., Timshel, P., De Vlaming, R., Abdellaoui, A., Ahluwalia, T.S., Bacelis, J., Baumbach, C., Bjornsdottir, G., Brandsma, J.H., Pina Concas, M., Derringer, J., Furlotte, N.A., Galesloot, T.E., Girotto, G., Gupta, R., Hall, L.M., Harris, S.E., Hofer, E., Horikoshi, M., Huffman, J.E., Kaasik, K., Kalafati, I.P., Karlsson, R., Kong, A., Lahti, J., Van Der Lee, S.J., Deleeuw, C., Lind, P.A., Lindgren, K.O., Liu, T., Mangino, M., Marten, J., Mihailov, E., Miller, M.B., Van Der Most, P.J., Oldmeadow, C., Payton, A., Pervjakova, N., Peyrot, W.J., Qian, Y., Raitakari, O., Rueedi, R., Salvi, E., Schmidt, B., Schraut, K.E., Shi, J., Smith, A.V., Poot, R.A.,

St Pourcain, B., Teumer, A., Thorleifsson, G., Verweij, N., Vuckovic, D., Wellmann, J., Westra, H.J., Yang, J., Zhao, W., Zhu, Z., Alizadeh, B.Z., Amin, N., Bakshi, A., Baumeister, S.E., Biino, G., Bonnelykke, K., Boyle, P.A., Campbell, H., Cappuccio, F.P., Davies, G., De Neve, J.E., Deloukas, P., Demuth, I., Ding, J., Eibich, P., Eisele, L., Eklund, N., Evans, D.M., Faul, J.D., Feitosa, M.F., Forstner, A.J., Gandin, I., Gunnarsson, B., Halldorsson, B.V., Harris, T.B., Heath, A.C., Hocking, L.J., Holliday, E.G., Homuth, G., Horan, M.A., et al. (2016). Genome-wide association study identifies 74 loci associated with educational attainment. *Nature* 533**,** 539-542.

Sarkar, S.N., Huang, R.-Q., Logan, S.M., Yi, K.D., Dillon, G.H., and Simpkins, J.W. (2008). Estrogens directly potentiate neuronal L-type Ca2+ channels. *Proceedings of the National Academy of Sciences* 105**,** 15148-15153.

Shen, X., Cox, S.R., Adams, M.J., Howard, D.M., Lawrie, S.M., Ritchie, S.J., Bastin, M.E., Deary, I.J., Mcintosh, A.M., and Whalley, H.C. (2018). Resting-state connectivity and its association with cognitive performance, educational attainment, and household income in the UK Biobank. *Biological Psychiatry: Cognitive Neuroscience and Neuroimaging* 3**,** 878-886.

Takeuchi, H., and Kawashima, R. (2021). Diet and Dementia: A Prospective Study. *Nutrients* 13**,** Article 4500.

Townsend, P. (1987). Deprivation. *Journal of social policy* 16**,** 125-146.

Veldsman, M., Kindalova, P., Husain, M., Kosmidis, I., and Nichols, T.E. (2020). Spatial distribution and cognitive impact of cerebrovascular risk-related white matter hyperintensities. *NeuroImage: Clinical* 28**,** 102405.
